# Parkinsonian Patients with Deficits in the Dysexecutive Spectrum are Impaired on Theory of Mind Tasks

**DOI:** 10.3233/BEN-129018

**Published:** 2013

**Authors:** Alberto Costa, Antonella Peppe, Matteo Martini, Katia Coletta, Massimiliano Oliveri, Carlo Caltagirone, Giovanni A. Carlesimo

**Affiliations:** ^1^IRCCS Fondazione Santa Lucia, RomeItaly; ^2^Dipartimento di Psicologia, University of Palermo, PalermoItaly; ^3^Department of Neuroscience, University of Rome Tor Vergata, RomaItaly

**Keywords:** Theory of Mind, Parkinson’s disease, executive functions

## Abstract

Understanding the mental states of others entails a number of cognitive processes known as Theory of Mind (ToM). A relationship between ToM deficits and executive disorders has been hypothesized in individuals with Parkinson's disease (PD). The present study was aimed at investigating the effect of dysexecutive deficits on ToM abilities in PD patients without dementia. Participants included 30 PD patients and 30 healthy subjects (HC). PD patients were divided into two groups according to their executive test performance: patients with poor (dysexecutive group; *n* = 15) and normal (executively unimpaired group; *n* = 15) performance. All participants were administered faux pas recognition written stories. The dysexecutive PD patients performed less accurately than both HC and executively unimpaired PD individuals on all faux pas story questions (*p* < 0.05); the executively unimpaired PD group performed as accurately as the HC group on the ToM tasks. Results of the study clearly demonstrate that PD is not tout court associated with ToM impairments and that these may occur in PD patients as a function of the degree of their executive impairment. Our findings also indirectly confirm previous data on the role of the prefrontal regions in mediating ToM capacities.

## 1. Introduction

The attribution of mental states, such as intentions and beliefs, to others has been called Theory of Mind (ToM) or mentalizing [1,2]. More specifically, it is assumed that ToM processes include both cognitive and affective components; the former refers to the ability to infer knowledge about another person's beliefs, and the latter to the ability to understand another's emotional states [3]. The cognitive and neural mechanisms of ToM functioning have been widely investigated in recent years. This is justified by the relevance that an impairment in ToM abilities may have on the quality of life of the affected individuals, in particular by interfering with their capacity to successfully interact with others [4]. Some neuropsychological investigations of individuals with brain injuries suggest that lesions involving frontal and temporo-parietal cortices are associated with ToM impairments [5–9]. These findings are supported by data from neuroimaging and transcranial magnetic stimulation studies indicating that a brain network including the prefrontal cortices (i.e. the dorsolateral and orbitofrontal cortices) the temporo-parietal

ISSN 0953-4180/13/$27.50 © 2013 – IOS Press and the authors. All rights reserved junction and the subcortical nuclei (i.e. the amygdala) underlies ToM processes [10–13].

Some behavioural studies have investigated ToM functioning in individuals with Parkinson's disease (PD). Indeed, PD is characterized early by a deficit of the dopamine brain pathways due to the primary loss of dopamine neurons within the substantia nigra and ventral tegmental area; the nigro-striatal dopamine circuitry is precociously affected by the disease, which subsequently also injures the mesocortical and mesolimbic pathways [14,15]. Consistent with this evidence, several studies in PD patients report the presence of a pattern of cognitive impairment characterized by poor performance on tests investigating the functional integrity of the frontal-striatal pathways [16–18], such as those tapping updating, highly demanding working memory abilities, and prospective memory functioning [16,19, 20]. It has also been found that ToM is impaired in PD patients. In fact, subsequent to Saltzman et al.'s [21] publication of the first data on this issue other studies have confirmed that PD patients perform worse than healthy controls across different ToM paradigms [22–25].

Moreover, the hypothesis of a relationship between ToM altered functioning and executive deficits has been advanced on the basis of the above evidence supporting both the dependency of ToM functioning on the integrity of the frontal lobes and early impairment of frontal-striatal-related cognitive abilities in PD patients. Indeed, in PD, the existence of a relationship between executive and ToM functioning was suggested first by Saltzman et al. [21] who found a significant correlation between ToM scores and some measures of executive abilities. In a subsequent study, Péron et al. [26] found that a subgroup of PD patients in the advanced stage of the disease (average duration of disease = 10.2 years) was significantly impaired on some cognitive aspects of ToM and that their performance on these measures correlated significantly with their score on the resistance to interference condition of the Stroop test. In a recent study by Santangelo et al. [23] in which PD patients without dementia were found to be more impaired on both cognitive and affective aspects of the ToM than healthy controls the authors reported a significant correlation between PD patients' performance on cognitive ToM measures and their score on the Frontal Assessment Battery. Yu et al. [27] obtained similar results in a more recent study. These findings seem to indicate a significant relationship between ToM abilities and executive functioning in PD patients. But the observation that all studies adopted a correlational design with relatively small sample sizes could limit the strength of this conclusion. In fact, in two different investigations in PD patients without dementia, Bodden et al. [28] and Roca et al. [29] failed to find a significant correlation between altered ToM capacities and various measures of executive functioning (e.g., Trail-Making test, verbal fluency, Modified Card Sorting Test). Correlational designs are also intrinsically limited because they do not allow inferring a causal relationship between variables. Indeed, a better understanding of the nature of the relationship between executive and ToM abilities could allow us to interpret ToM deficits in the PD population and could provide greater knowledge about ToM mechanisms per se.

The aim of the present study was to investigate whether the presence of ToM impairments in PD patients depends specifically on cognitive deficits in the executive domain. To overcome the limits of correlational designs, we recruited individuals suffering from PD without dementia who presented with an executive domain deficit, PD patients without cognitive impairments, and a group of healthy controls (HC). The effect of executive deficits on ToM abilities was investigated by analyzing between-group differences in their performance on faux pas recognition tasks, a paradigm previously shown to be sensitive to PD [26,29]. We predicted that if ToM alterations are specifically associated with executive impairments in PD then only executively impaired patients (and not PD patients without executive deficits) should perform worse than healthy controls on ToM tasks.

## 2. Methods

### 2.1. Participants

Thirty individuals with idiopathic PD and 30 normal controls participated in this study after giving their informed consent. The diagnosis of idiopathic PD was made by an expert neurologist based on the following criteria 1) presence of at least two of the four cardinal parkinsonian symptoms; and 2) good chronic response to L-dopa treatment. Exclusion criteria included the following: 1) dementia based on the Diagnostic and Statistical Manual of Mental Disorders criteria for dementia (American Psychiatric Association, 1994) and a Mini Mental State Examination score $< 26$ [30,31]; 2) a pathological performance on one test of the neuropsychological battery investigating cognitive functions outside the executive domain (below is a descrip-

Table 1
Socio-demographic and clinical characteristics of participants in the PD and HC groups

| | Dysexecutive PD group $N = 15$ | Executively unimpaired PD group $N = 15$ | Healthy controls | F value | p level |
|---|---|---|---|---|---|
| Gender (F/M) | 5/10 | 4/11 | 19/11 | | |
| | | Mean (SD) | | | |
| Age (years) | 62.8 (5.3) | 62.1 (6.7) | 64.2 (8.3) | 0.46 | $> 0.60$ |
| Years of formal education | 8.3 (3.2) | 9.1 (3.5) | 9.6 (3.6) | 0.73 | $> 0.40$ |
| MMSE score | 27.3 (0.9) | 28.5 (1.9) | 27.9 (1.9) | 1.79 | $> 0.10$ |
| UPDRS score | 30.1 (10.2) | 27.3 (14.7) | | 0.36 | $> 0.50$ |
| Years of disease duration | 7.8 (4.7) | 6.4 (4.7) | | 0.66 | $> 0.40$ |
| Age at disease onset | 55.0 (7.4) | 55.7 (9.5) | | 0.05 | $> 0.80$ |
| Beck Depression score | 18.6 (11.1)* | 9.7 (6.8) | 9.4 (6.4) | 7.49 | $= 0.001$ |

MMSE = Mini Mental State Examination; UPDRS= Unified Parkinson's Disease Rating Scale.

tion of the neuropsychological test battery used for cognitive screening); 3) presence of severe systemic or metabolic diseases; 4) marked cortical and subcortical atrophy and/or ischemic vascular lesions on CT and/or MRI scans; 5) history of other neurological or psychiatric disorders, head trauma, or substance abuse; vi) severe functional impairment of the autonomic nervous system. To quantify extrapiramidal symptom severity, PD patients were administered the Unified Parkinson's disease Rating Scale-Part III [32]. At the time of the assessment, all patients were taking dopamine therapy with levodopa and/or dopamine agonists. No one of the PD patients participating to the study underwent surgical intervention for deep brain stimulation.

According to the study design, to investigate the relationship between executive and ToM impairments in PD the patients were split into two subgroups according to the following parameters: 1) a pathological score corresponding to a performance $<$ 95% of the lower tolerance limit of the normal population distribution on the Modified Card Sorting test (MCST; see below for a detailed description of the this test [33]); *ii*) 2) a score below the median of the whole PD group on at least two of the other tests individually examining the following executive subcomponents, according to the analysis by Miyake et al. [34] and Fisk and Sharp [35]: inhibition (Stroop Test [36]), access to mental representations (Phonemic Fluency Test) [37]; attentional set-shifting (Trail Making Test) [38]; updating (Digit Span Backward) [39] (see below for a detailed description of all these tests). We chose to adopt the MCST as a cardinal measure to classify PD patients as dysexecutive because it requires different executive abilities, particularly set-maintenance, set-shifting, updating, and abstract reasoning. In fact, it has been found sensitive to cognitive impairment in PD patients without dementia [40, 41] and is recommended by the Movement Disorder Society Task Force to assess executive functioning in PD patients [31]. Moreover, card- sorting paradigms have also been found significantly related to the activity of the frontal-striatal circuitries in individuals with PD [42].

Most HC participants were recruited from PD patients' relatives. Exclusion criteria for the HC group were head trauma, history of psychiatric disease, central nervous system disease, severe systemic disease, taking drugs or medications that can affect cognitive performances.

The clinical characteristics of the PD groups and the socio-demographic characteristics of all groups are reported in Table 1.

### 2.2. *Neuropsychological test battery*

The neuropsychological battery included tests that evaluate the following cognitive domains: episodic memory (Immediate and Delayed Recall of a 15-Word List; Immediate and Delayed Prose Recall) [37, 43]; short-term memory (Digit Span Forward and Corsi Block Tapping test Forward) [44]; abstract reasoning (Raven's Coloured Progressive Matrices) [37]; constructive praxis (Copy of Drawings and Copy of Drawings with Landmarks) [37]; executive functions (MCST [33]; The Stroop test [36]; the Trail Making Test [38]; Digit Span Backward [39]; Phonemic Fluency Test [37]). Published normative data for score adjustment according to age, education and gender as well as normality cut-off scores (corresponding to a performance $\geqslant$ 95% of the lower tolerance limit of the normal population distribution) were available for all tests.

### 2.3. *Tests of executive functions*

#### 2.3.1. *The Modified Card Sorting Test*

The MCST [33] consists of four stimulus cards; each has a unique colour, shape and number of items. The response cards consist of two sets of 24 cards; each card has one attribute in common with each of the stimulus cards. The subject has to sort the cards according to colour, shape or number. Three separate scores are computed for number of criteria achieved (range: 0–6; cut-off score: 4.25), number of perseverative errors (i.e. when the patient persists with a category even after being told it is incorrect) and number of non-perseverative errors. To classify PD patients as executively impaired and executively unimpaired, we used only the score referring to categories achieved.

### 2.3.2. *The stroop test*

The stroop test [36] is commonly used to assess dysexecutive deficits in neurological populations [42]. It includes three subtests; each consists of a sheet of paper displaying 100 stimuli, regularly aligned in ten columns and ten rows. The subject's task is to check the paper as quickly as possible starting from the left upper board and proceeding vertically down to the tenth column on the right. In the first subtest (word-reading subtest), the subject has to read the words representing the names of five colours (red, blue, green, brown and violet). In the second subtest (naming-colours subtest), the subject is shown coloured squares (i.e., the above colours) and is asked to name their colours. In the third subtest, written words representing the above colours are shown in conflicting colours (e.g., the word red written in blue ink) and the subject has to name the ink colour of the printed word but not read the word (resistance to interference subtest). To evaluate performance, response times and correct answers were recorded.

*Trail making test* (TMT) [43]. The TMT is a visuomotor task that assesses cognitive flexibility as well as motor speed and coordination. Time to complete the TMT-A (i.e. tracking with pencil lines that join numbers in increasing order and the TMT-B (i.e. tracking lines that alternatively join numbers in increasing order and letters in alphabetical order) were recorded. Another measure was obtained by subtracting the time needed to complete the TMT-A from the time needed to complete the TMT-B (TMT B-A). Only the latter measure was used to evaluate subjects' performance since it allow to quantify the specific cost for the implementation of shifting processes.

*Digit span backward* [39]. Subjects are instructed to repeat backward strings of digits (varying in length from 3 to 9 digits) read by the examiner. The score is the longest sequence reproduced correctly (score range: 0–9).

*Phonemic fluency test* [37]. In this test, the subject is required to generate words beginning with the letters "A", "F" and "S" in three different trials, each lasting 60 seconds.

To obtain a normalized measure to evaluate PD patients' performance on the Stroop test, the Trail Making test, the Digit Span Backward and the Phonemic Fluency tests, Z-scores were computed by considering mean and DS values of healthy controls (if available data from the normative population were used. In the other cases, i.e., for the Digit Span Backward and the Stroop test, data from the control group participating in the present study were used).

## 2.4. *Theory of Mind procedure*

To evaluate ToM abilities we adopted a slightly revised version of an experimental procedure we used in a previous study [10]. This procedure was constructed basing on previous ToM paradigms [46,47]. We composed 12 written short stories followed by a series of questions. The stories were composed of six Recognition of Faux Pas tasks and six control stories. Participants had to read the stories presented at the centre of a computer screen and then answer some questions by choosing among different alternatives. There was no time limit for reading the story. Subjects told the examiner when they were ready and than began to answer the questions.

In faux pas stories a prior event that occurred between two people is described. Subsequently, the two characters meet again and one, forgetting the prior encounter, says something awkward, which could offend the other person. The questions require the subject to detect the faux pas (question 1: "Did someone say something they shouldn't have said?"), to understand the mental state of the listener (question 2: "Why shouldn't they have said it?") and to understand the mental state of the speaker (question 3: "Why did they say it?"), choosing between different alternatives by pressing the corresponding button on the keyboard. Control stories were identical to the faux pas stories, but no faux pas occurred between the characters. In the control tasks, the subjects had to answer the following question: "Did someone say something they shouldn't have said?". For all faux pas and control stories, accuracy and response times were recorded separately for each question. A score of 1 was assigned to correct answers and a score of 0 to wrong answers. The time the subjects took to read the stories was also recorded. Moreover, following indications from previous studies [9,45], a faux pas composite score was computed by summing the scores a subject obtained on the three ToM questions and the control story questions (i.e., correct rejection of faux pas). Then, the total score was divided by 24 (i.e. the number of questions).

Each subject was administered the stories in one experimental session that lasted about 30 minutes. The occurrence of faux pas and control stories was interblended.

### 2.5. Depression symptom assessment

The potential negative influence of depressive disorders on cognitive performance was previously reported [40,48]. Therefore, we evaluated the severity of depressive symptoms by administering the Beck Depression Inventory (BDI), a self-administered instrument that has shown good reliability in evaluating severity of depression in PD [49]. We administered the cognitive tests, the BDI and the ToM experimental tasks on two different days.

### 2.6. Statistical analyses

Preliminary One-way ANOVAs were applied to sociodemographic and clinical data and BDI scores. A general MANCOVA (Roy's Root) with the BDI score as a covariate was performed to compare the overall performance of the two PD sub-groups on the tests of the neuropsychological battery (with the exception of tests investigating executive functions). A further MANCOVA was performed to compare the three experimental groups on the tests investigating individual executive subcomponents.

We performed two Mixed ANCOVAs with Group (dysexecutive PD group vs. executively unimpaired PD group vs. HC) and Question (question 1 vs. question 2 vs. question 3) as within factor to analyze accuracy and response times on faux pas tasks. One-way ANCOVAs were executed to compare Group accuracy and response times on control stories and time taken to read the two types of stories. Then, an ANCOVA was performed to compare the faux pas composite score between the three groups.

In the case of significant effects of main factors or interactions, post-hoc HSD-Tukey tests were performed. For all analyses involving ToM and neuropsychological tests, the BDI score was inserted as covariate.

## 3. Results

Because of their pathological score on the MCST (i.e. categories achieved) and a performance score below the group median on at least two of the other tests examining the different executive subcomponents, 15 PD patients were classified as dysexecutive (categories achieved: mean = 2.0; SD = 0.6; perseverative errors: mean = 11.1; SD = 7.1) and 15 PD patients as executively unimpaired (categories achieved: mean = 5.0; SD = 0.8; perseverative errors: mean = 2.6; SD = 2.7). Performance scores of individuals in the two PD subgroups on the executive function tests are reported in Table 2. The MANCOVA performed to compare the three experimental groups on neuropsychological tests investigating the executive subcomponents showed a significant effect ($F(2,56) = 4.57$; $p < 0.01$). Post-hoc analyses confirmed that individuals in the dysexecutive group had reduced executive abilities as showed by their lower performance than HC on the Stroop test (errors committed: $p = 0.06$; response times: $p = 0.01$) and on the TMT ($p < 0.001$). Moreover, the performance of the disexecutive PD group was also significantly lower than that of the executively unimpaired PD group on the TMT ($p < 0.05$). The same analyses also confirmed that PD patients belonging to the executively unimpaired group had not reduced executive capacities in respect to HCs as shown by the absence of significant between groups differences on above tests (all p consistently $> 0.10$).

As shown in Table 1, the three groups were comparable in age, years of formal education and MMSE score and the two PD groups did not differ on various clinical variables such as UPDRS score and disease duration. However, the executively impaired PD patients obtained higher scores on the BDI than both HC ($p < 0.01$) and executively unimpaired PD individuals ($p < 0.01$), whereas no significant difference was found between the two latter groups on this scale ($p > 0.90$). The general MANCOVA applied to the neuropsychological test scores revealed no significant difference between the two PD subgroups ($F(9,19) = 1.28$; $p > 0.30$). Patients' performance scores on the neuropsychological tests are reported in Table 3.

## 4. Theory of mind tasks

### 4.1. Faux pas accuracy

The performance accuracy of the individuals in the three groups on the three faux pas questions are illustrated in Fig. 1 (panel a). The mixed ANCOVA performed on their scores on the three questions shows a significant main effect of the factors Group (F(2,56)

Table 2
Table shows average performance scores obtained by PD patients on the tests examining individual executive subcomponents. Values are reported as Z scores

| | | Dysexecutive group | Executively unimpaired group | Median value of the whole PD sample |
|---|---|---|---|---|
| | | Mean (SD) | | |
| Stroop test Resistance to interference subtest | Response times | −1.48 (1.99) | −0.45 (1.6) | −0.89 |
| | Errors | −3.01 (3.8) | −0.71 (1.7) | −1.53 |
| Trail Making Test Part B-Part A | Response times | −1.34 (1.5) | −0.43 (0.6) | −0.57 |
| Digit Span Test Backward | Accuracy | −0.16 (0.5) | −0.10 (0.7) | −0.16 |
| Phonemic Fluency | Accuracy | −0.17 (0.43) | −0.12 (0.8) | −0.55 |

Table 3
Performance of PD patients (i.e. average scores and percentage of pathological values) on the neuropsychological test battery

| Neuropsychological Tests | Dysexecutive PD group | Executively unimpaired PD group |
|---|---|---|
| **Mnesic functions** | Mean (SD) | Mean (SD) |
| *Short-term memory* | | |
| Digit span forward | 4.8 (0.9) | 5.5 (0.8) |
| Corsi test forward | 4.3 (0.6) | 5.0 (0.7) |
| *Episodic memory* | | |
| Word list recall-immediate | 36.2 (6.2) | 38.8 (8.5) |
| Word list recall-delayed | 7.4 (2.2) | 7.9 (2.5) |
| Prose recall-immediate | 5.4 (1.4) | 5.6 (0.7) |
| Prose recall-delayed | 5.2 (1.4) | 5.5 (0.9) |
| **Visual-spatial abilities** | | |
| Free hand copying of drawings | 8.3 (1.1) | 9.2 (1.5) |
| Copying drawings with landmarks | 64.5 (4.6) | 66.5 (3.1) |
| **Abstract reasoning** | | |
| Raven's Progressive Matrices 47 | 24.2 (4.8) | 28.5 (3.6) |

For each test, a high score indicates better performance.

= 3.5; $p$ = 0.036) and Question (F(2,114) = 92.9; $p < 0.001$), whereas the Group*Question interaction did not approach statistical significance (F(4,114) = 1.88; $p > 0.10$). Post-hoc tests revealed that the dysexecutive PD individuals (mean = 3.44; SD = 1.1) were significantly less accurate than both HC (mean = 4.31; SD = 1.1; $p$ = 0.020; Cohen's $d$ [50] = 0.79) and executively unimpaired PD subjects (mean = 4.4; SD = 1.2; $p < 0.01$; Cohen's $d$ = 0.87). Moreover, all groups obtained lower average scores on the third question (mean = 2.6; SD = 1.5) than on the first (mean = 5.0; SD = 1.1; $p < 0.001$) and second (mean = 4.7; SD = 1.0; $p < 0.001$) questions, respectively. Responses to the first question only tended to be more accurate than those to the second question ($p$ = 0.010).

As for the between-group comparison on the faux pas composite score, the Group effect was significant (F(2,56) = 3.53; $p$ = 0.036). Post-hoc HSD-Tukey tests show that the composite score was significantly lower in the dysexecutive PD group (Mean = 0.61; SD = 0.12) compared with both HC (Mean = 0.75; SD = 0.12; $p < 0.01$; Cohen's $d$ = 1.17) and the executively unimpaired PD group (Mean = 0.75; SD = 0.15; $p$ = 0.011; Cohen's $d$ = 1.03). No significant difference was found for this measure between the executively unimpaired PD group and HC ($p > 0.90$).

### 4.2. *Faux pas response time*

Response times for correct answers on faux pas stories are reported in Fig. 1 (panel b). Analysis of time needed to read the stories revealed no significant between-group difference (dysexecutive PD group: mean = 40244 ms; SD = 13932; HC: mean = 31975 ms; SD = 14973; executively unimpaired PD group: mean = 36046; SD = 15755) as shown by the lack of significance of the Group effect (F(2,56) = 1.30; $p > 0.20$).

Regarding subjects response latencies on the three questions, the lack of significance of the Group effect (F(2,56) = 0.18; $p > 0.40$) and the Group*Question interaction (F(4,114) = 1.24; $p > 0.20$) shows that the dysexecutive PD patients (mean = 11864 ms; SD = 7739) were as fast as HC (mean = 8983 ms; SD = 3904) and executively unimpaired PD patients (mean = 10448; SD = 3104) in responding to faux pas questions. Nevertheless, the main effect of Question was significant (F(2,114) = 65.3; $p < 0.001$). Post-hoc

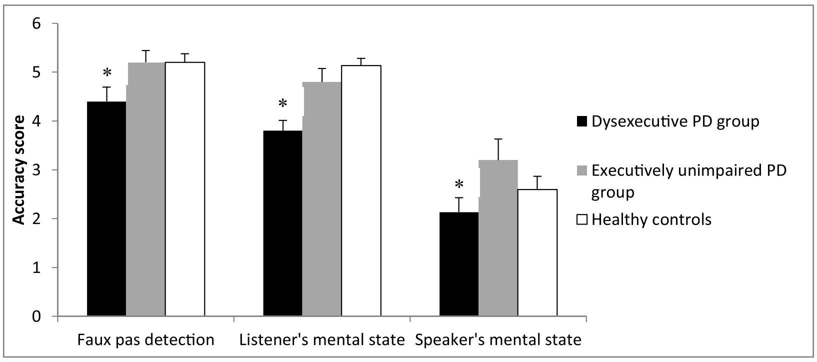

**Panel a**

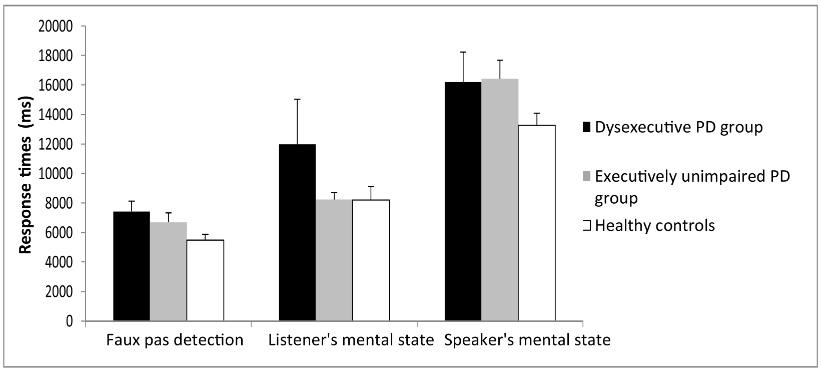

**Panel b**

Fig. 1. The Figure illustrates accuracy (panel a) and response times (panel b) of the subjects in the PD and HC groups on the faux pas recognition tasks. *Indicates a significant difference between the dysexecutive PD group and the two other experimental groups.

HSD Tukey tests document that all participants in the three groups were faster in responding to the first question (mean = 6269 ms; SD = 2518) than to the second (mean = 9156 ms; SD = 7168; $p < 0.001$) and third (mean = 14781 ms; SD = 5832; $p < 0.001$) questions, and that their response times to the second question were significantly lower than those to the third question ($p < 0.001$).

### 4.3. Control stories

The analysis performed on the accuracy score of the control stories showed no main effect of Group (F(2,56) = 0.46; $p > 0.60$). In fact, the three experimental groups performed comparably on these stories (dysexecutive PD group: mean = 3.3; SD = 1.6; HC: mean = 4.1; SD = 1.3; executively unimpaired PD group: mean = 3.7; SD = 1.9).

Similar results were found by analysing response times. In fact, no significant between-group difference was found for either time needed to read the stories (F(2,56) = 0.23; $p > 0.70$) or response times for answering the story question (F(2,56) = 0.43; $p > 0.60$).

## 5. Discussion

The present study was aimed at investigating the influence of executive deficits on ToM functioning in PD patients without dementia. According to the previously documented relationship between frontal lobes activity and ToM [11–13] and the reported significant correlation between performance on executive and ToM tasks in PD patients [21,23,27], we predicted that reduced ability to perform ToM tasks would be found in PD individuals with executive disorders. The results of the study confirmed our prediction, indicating that in the PD population reduced ToM abilities were clearly related to the presence of executive impairments. This conclusion is confirmed by two orders of evidence. First, PD patients who exhibited a dysexecutive cognitive profile, which was documented by their pathological performance on the Modified Card Sorting Test and their score below the PD group median on other measures of executive subcomponent functioning (i.e., Stroop test, Trail Making test, Phonemic Fluency test and Digit Span Backward), were less accurate than both HC and PD patients without executive impairments on the ToM tasks (i.e. all faux pas story questions). Second, executively unimpaired PD patients performed as accurately as HC on the ToM tasks. The fact that participants in the dysexecutive PD group did not have longer response times than participants in the other groups and that no significant difference was found between the two PD sub-groups in overall performance on the tests of the neuropsychological battery makes it unlikely that the poor ToM performance exhibited by the dysexecutive PD group was due to a generalized cognitive impairment or global reduction of attentional resources.

Although (as mentioned above) several authors have documented decreased ToM abilities in individuals with PD [24,25], findings on the relationship between executive functions and ToM abilities in the PD population are still controversial. In fact, some authors [21, 23] reported a significant association between PD patients' performance on the two kinds of tasks, whereas others failed to find such an association [28,29]. Some methodological factors could account for this incongruence: first, the different clinical characteristics of the PD samples recruited; second, the heterogeneity of the instruments administered to investigate ToM and executive abilities across the different studies; and third, the possible influence of neuropsychiatric symptoms, such as apathy, that, in PD population, has been shown to be associated to ToM deficits [23]. Moreover, as all these studies adopted correlational designs, it is impossible to explore the nature of the relationship between ToM and executive processes. In fact, as pointed out in a recent paper by Aboulafia-Brakha et al. [51], this issue has to be clarified in various neurological populations. In particular, in their review these authors report the existence of a parallel impairment in ToM and executive function tasks in 22 out of 24 studies examined, thus supporting a strong association between the two domains [51]. Nevertheless, a few studies that investigated neurological populations demonstrated that ToM abilities can be preserved in patients with dysexecutive impairments [52,53], as well as the reverse pattern of performance, that is, in one subject, preserved executive functions and impaired ToM reasoning [54]. The multifaceted features of the two constructs could account for the difficulty in clarifying their relationship. Specifically, the fact that not all published studies addressed all the subcomponents of executive abilities or of ToM processes could limit interpretation of the results. This might be particularly true for those studies with null findings. However, some authors suggest that a functional dependency exists between executive functions and ToM capacities [55,56]. In particular, Fish and Happe [56] found significant ToM improvement in autistic children after they were submitted to cognitive training focused on the implementation of executive abilities. The results of the present study could be interpreted in this vein. Indeed, the ToM tasks used here require the subject to implement various operations that might be altered in patients with dysexecutive deficits. In particular, the faux pas recognition paradigm is considered a complex task in which subjects have to represent at least two mental states, namely, that the person making the faux pas does not know he should not say it (cognitive representation) and that the person hearing it would feel insulted or hurt (affective representation) [3, 57]. Therefore, high level abilities such as those required to inhibit the self-perspective, to integrate information deriving from a past event and from the other and to flexible access to different mental representations are strongly involved in this task and may well be affected by the occurrence of executive deficits [58]. In fact, among ToM paradigms the faux pas is the one most closely associated with executive performance in neurological populations [51]. To further investigate whether a functional dependency exists between executive impairment and ToM altered functioning in PD patients, further studies are needed to investigate the effect of rehabilitative training of executive functions on ToM abilities [56].

Some studies have reported the presence of a differential deficit of the cognitive vs. the affective component of ToM, suggesting that the former might be more impaired in these patients [26,29]. Although the findings of other studies do not support this dissociation [23,28], some data also suggest a specific correlation between the cognitive component of ToM tasks and executive performance of these patients [23,26]. In our study, we did not directly look for a possible differential impairment of the cognitive and affective aspects of ToM as a function of the executive impairment. In fact, as discussed above, the faux pas recognition paradigm likely entails both cognitive and affective elaborations [3,57]. In fact, the dysexecutive PD patients who participated in the present study were less accurate than both HC and the executively unimpaired PD patients in responding to all faux pas questions including those investigating the ability to detect that the listener could be hurt (affective component), as well as the one that requires understanding the motivation/intention of the speaker in making the faux pas (cognitive component). Therefore, we can argue that the difficulty exhibited by dysexecutive PD patients in recognizing the faux pas is related to their failure to process purely affective or cognitive aspects of the task, or both.

The finding that only PD patients with dysexecutive deficits show reduced ToM functioning indirectly confirms that altered frontal lobe activity might explain the performance of these individuals. In fact, the Modified Card Sorting Test and the additional executive tests we administered to the PD patients are reported to be sensitive to altered frontal functioning in PD individuals [17, 42]. Indeed, various regions of the prefrontal cortex, including the dorsolateral and orbito-frontal portions and the anterior-cingulate cortex, seem to be involved in the different aspects of these tests [17,59,60]. Activity in these regions is affected by PD depending on the progression of dopamine depletion in the frontal-striatal pathways [16,18]. More specifically, dopamine depletion in PD primarily involves the rostrodorsal extent of the head of the caudate nucleus, a region strongly connected to the dorsolateral prefrontal cortex, and only later affects the more ventral parts of this structure, which are preferentially connected to the ventral prefrontal cortex [14,15]. Alterations of functioning of the frontal-striatal networks are reported to be associated with executive deficits in PD patients [17,60]. Thus, our findings are in line with previous behavioural [8, 12,13,61], neuroimaging [62,63] and transcranial magnetic stimulation [10,11] data that indicate the critical involvement of some regions of the prefrontal cortex in ToM. It should be noted, however, that the brain correlates of ToM as well as the relationship between dopamine and ToM functioning have been poorly explored in PD patients. In this regard, data from Peron et al.'s study [26] show no significant effects of dopamine compound administration/withdrawal on various ToM measures in these patients. More in general, in PD patients, the relationship between dopamine administration and cognitive functioning is not linear insofar it has been reported to improve, negatively affect or leave unchanged subjects' performance [16,17]. Unfortunately, all PD individuals participating in our study were evaluated while taking their usual dopamine therapy and we did not include a control condition (i.e., after a dopamine therapy wash-out period- "off" condition) to evaluate the effect of dopamine on ToM. To further explore these issues, neuroimaging studies in PD subjects are needed in which dopamine pharmacotherapy is manipulated.

In conclusion, the results of the present study indicate that ToM is not tout court impaired in individuals with PD but is specifically related to cognitive impairments within the executive domain. This is important information from a clinical point of view. For example, individuals with reduced ToM abilities might have difficulty in successfully interacting with others [4], which would negatively affect their social activities and interpersonal satisfaction. This could alter the psychological and affective functioning of these patients and thus contribute to a significant reduction of their quality of life [23]. These observations clearly indicate the importance of assessing psychopathological symptoms in PD patients. Particularly in patients with dysexecutive deficits, this assessment should include an accurate investigation of ToM disorders to plan for better therapeutic interventions.
